# Attitudes, Beliefs and Knowledge About Brain Donation Among Black Americans; Focus Group Feedback Results

**DOI:** 10.1002/alz.093520

**Published:** 2025-01-09

**Authors:** Allison M Caban‐Holt, Michael L. Cuccaro, Takiyah D. Starks, Ashley Gregory, Larry D. Adams, Jenny Chavez, Paris Pryor‐Banks, Shawnta' Lloyd, Jonathan L. Haines, Christiane Reitz, Jeffery M. Vance, Tina Duke, Mary E Douglas, Margaret A. Pericak‐Vance, Goldie S. Byrd

**Affiliations:** ^1^ Maya Angelou Center for Health Equity, Wake Forest University School of Medicine, Winston‐Salem, NC USA; ^2^ John P. Hussman Institute for Human Genomics, Miami, FL USA; ^3^ Gertrude H. Sergievsky Center, Taub Institute for Research on the Aging Brain, Departments of Neurology, Psychiatry, and Epidemiology, College of Physicians and Surgeons, Columbia University, New York, NY USA; ^4^ Department of Population and Quantitative Health Sciences, Case Western Reserve University, Cleveland, OH USA

## Abstract

**Background:**

African Americans (AA) are underrepresented in Alzheimer’s disease (AD) brain donation research, making up approximately 2% of brain donations to the National Alzheimer’s Coordinating Center (NACC). Focus groups were conducted to obtain qualitative information to expand upon survey data that was collected previously to gain additional insights into the attitudes of Black∖AA individuals toward brain donation and perceptions of medical research.

**Method:**

A brain donation focus group facilitator guide was created based upon earlier survey findings. Three focus groups of Black/AA adults were conducted in each of the following states: Florida, New York, North Carolina, and Ohio, United States between September and December, 2023. Sessions were audio recorded and written notes were also produced. Discussion topics posed to focus group participants included their preferred sources of health information, knowledge about AD, brain donation processes, motivations for and barriers to research participation

**Result:**

Across all locations, 83 Black∖AAs participated in focus groups, females = 80.72%, with an average age = 66.13 years, and average education = 13.88 years. Preliminary review of focus group responses suggest that 1) awareness of brain donation research procedures and processes is low in the general population that is not involved in research, 2) most participants did not know that brain donation was not covered by organ donation through one’s driver’s license, 3) most assumed that brain donation would be disfiguring and would preclude having an open casket funeral, 4) few participants had been approached about brain donation for themselves or a family member, 5) most participants were open to learning more about brain donation, 6) participants expressed interest in the research being focused on and conducted by AAs, which they suggested was progress from the past, 7) religion was not expressed as a significant factor in consideration of brain donation, however, 8) family agreement was voiced as important in this decision‐making process.

**Conclusion:**

These insights will be used to create a community‐informed educational program for AA∖Black communities about AD research and brain donation that will focus on increasing knowledge of brain donation procedures, focus on family engagement in research, and provide opportunities for participation.

